# Litter Decomposition in a Semiarid Dune Grassland: Neutral Effect of Water Supply and Inhibitory Effect of Nitrogen Addition

**DOI:** 10.1371/journal.pone.0162663

**Published:** 2016-09-12

**Authors:** Yulin Li, Zhiying Ning, Duo Cui, Wei Mao, Jingdong Bi, Xueyong Zhao

**Affiliations:** Cold and Arid Regions Environmental and Engineering Research Institute, Chinese Academy of Sciences, 320 Donggang West Road, Lanzhou 730000, China; Tennessee State University, UNITED STATES

## Abstract

**Background:**

The decomposition of plant material in arid ecosystems is considered to be substantially controlled by water and N availability. The responses of litter decomposition to external N and water, however, remain controversial, and the interactive effects of supplementary N and water also have been largely unexamined.

**Methodology/Principal Findings:**

A 3.5-year field experiment with supplementary nitrogen and water was conducted to assess the effects of N and water addition on mass loss and nitrogen release in leaves and fine roots of three dominant plant species (i.e., *Artemisia halondendron*, *Setaria viridis*, and *Phragmites australis*) with contrasting substrate chemistry (e.g. N concentration, lignin content in this study) in a desertified dune grassland of Inner Mongolia, China. The treatments included N addition, water addition, combination of N and water, and an untreated control. The decomposition rate in both leaves and roots was related to the initial litter N and lignin concentrations of the three species. However, litter quality did not explain the slower mass loss in roots than in leaves in the present study, and thus warrant further research. Nitrogen addition, either alone or in combination with water, significantly inhibited dry mass loss and N release in the leaves and roots of the three species, whereas water input had little effect on the decomposition of leaf litter and fine roots, suggesting that there was no interactive effect of supplementary N and water on litter decomposition in this system. Furthermore, our results clearly indicate that the inhibitory effects of external N on dry mass loss and nitrogen release are relatively strong in high-lignin litter compared with low-lignin litter.

**Conclusion/Significance:**

These findings suggest that increasing precipitation hardly facilitates ecosystem carbon turnover but atmospheric N deposition can enhance carbon sequestration and nitrogen retention in desertified dune grasslands of northern China. Additionally, litter quality of plant species should be considered when modelling the carbon cycle and nutrient dynamics of this system.

## Introduction

The decomposition of plant material in arid and semiarid ecosystems plays an important role in regulating carbon (C) storage and nutrient cycling because global drylands account for approximately 20% of the soil organic carbon pool [1]. The rate at which plant material decomposes is considered to be substantially controlled by water and nitrogen (N) availability in the soils of arid and semiarid ecosystems. For example, Yahdjian [2] found that litter decomposition rates were positively correlated with incoming annual precipitation in a semiarid steppe, and Liu et al. [3] observed that additions of N stimulated the rate of litter decomposition in a semiarid grassland. However, some evidence demonstrated few or no relationships between litter decomposition and changes in N and water availability in arid regions, suggesting that the responses of litter decomposition to changes in N and water availability varied at the local scale. To accurately predict C storage and N cycling in terrestrial ecosystems, it is essential to determine how litter decomposition responds to water and N availability in arid and semiarid regions, particularly under the current increase in N input and inter-annual precipitation variability induced by human activities and global warming [4].

In most biomes, litter decomposition and subsequent nutrient loss are driven primarily by microbial activity [5]. Any environmental factor that affects soil microbial activity will eventually control the rate of decomposition [6]. Consequently, N and water availability in arid and semiarid ecosystems are considered primary influences of litter decomposition because decomposer microbes require N and water from the surrounding environment [7, 8]. Some previous observations have suggested that litter decomposition rates are well correlated with precipitation or actual evapotranspiration in arid regions [3, 6, 9]. Rainfall also enhances mass loss by facilitating leaching of water-soluble compounds [10] and breakdown of surface litter in the initial stage of decomposition [11]. However, several lines of opposing evidence suggested that litter decomposition is not correlated with seasonal or annual precipitation in arid and semiarid ecosystems [12–14]. For example, no significant correlation was found between the total rainfall and total mass losses in the Judean desert [13]. A recent study in the Chihuahuan Desert suggested that decomposition rates did not respond to altered precipitation until after 19 months in a 41-month incubation [15].The results about how soil N availability effect on decomposition have also been controversial. Using a meta-analysis approach, Knorr et al. [16] reported stimulatory, neutral, or suppressive effects of external N on litter decomposition across grassland, forest or tundra ecosystems. The observed positive, neutral, or negative responses of litter decomposition to either N addition or water availability warrant further investigation.

The effects of increasing N and water on the decomposition of aboveground litter have been investigated extensively in most biomes, although conflicting responses of litter decomposition to N and water availability have been observed. However, knowledge of how N and water availability alters root decomposition is very limited. Some previous studies have suggested that root decomposition differs from that of aboveground litter in temperate ecosystems. For example, Austin et al. [8] observed no response of aboveground decomposition to water pulse content, whereas belowground decomposition was significantly altered by water pulses. Additionally, a study in a semiarid grassland in northern China suggested that the effect of rainfall on aboveground decomposition was small, whereas belowground decomposition was more dependent on inter-annual rainfall variability [17]. The differences in litter chemistry and differences in microbial decomposer communities on the surface and in the soil likely affects litter decomposition at different positions [18, 19]. In addition, root litter in mineral soil may be subject to milder extremes of temperature and moisture compared to aboveground litter, particularly in arid regions where mineral soil buffers root litter from moisture oscillations following rainfall pulses [20]. These possibilities may contribute to the different responses of litter decomposition to water and N availability between leaves and roots and thus call for comprehensive studies of such responses in arid regions.

In the semiarid dune grasslands of northern China, severe land desertification and subsequent wind erosion carry away substantial amounts of clay and silt particles and lead to soil coarsening and impoverishment, resulting in decreased soil N availability and soil water holding capacity [21, 22]. Poor soil N and water availability are therefore considered the primary factors controlling ecosystem processes in this area. To examine how N and water availability affect litter decomposition and nitrogen release in desertified semiarid dune grassland ecosystems, dry mass loss and nitrogen dynamics were determined in the leaf litter and fine roots of three dominant species (i.e., *Artemisia halondendron*, *Setaria viridis*, and *Phragmites australis*) with contrasting litter chemistry in a 3.5-year field incubation experiment with N and water addition. Our objectives were to determine 1) how N and water addition and their interactions affect dry mass loss and nitrogen release of fine roots and leaf litter and 2) whether the responses of decomposition rates to water and N addition differ between leaf and root litter. Our underlying hypotheses were the following: adding N and water singly or simultaneously will stimulate mass loss and nitrogen release in roots and leaves because N and water availability are considered the primary limiting factors in severely desertified dune grasslands; root litter will decompose faster than leaf litter due to the lower frequency of moisture oscillations in the soil [20].

## Materials and Methods

### Study site

**Fig 1:**
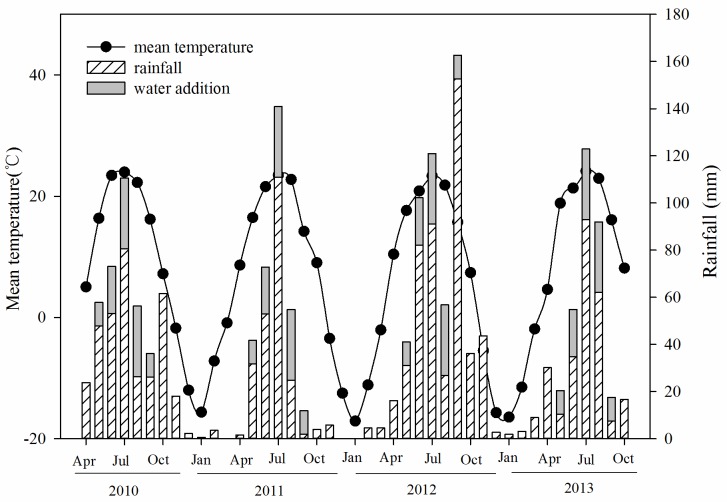
Microclimatic conditions during the experimental period from 2010 to 2013 at the study site: natural rainfall, artificial rainfall (water addition) and mean temperature.

The study was conducted in a severely desertified dune grassland near Naiman Desertification Research Station, Chinese Academy of Sciences (N 42°55′ and E 120°41′, 350 m above mean sea level). The study site is located in the southwest of Horqin Sand Land, eastern Inner Mongolia, China, in a continental semi-arid monsoon climate zone. The average annual temperature is 6.4°C, with monthly averages ranging from a minimum of -13.1°C in January to a maximum of 23.7°C in July. The yearly accumulated air temperature above 10°C ranges from 3000 to 3400°C. The frost-free period lasts 137–150 days per year. The mean annual precipitation is 362 mm, nearly 80–90% of which falls from April through October. During the period of the experiment from 2010 to 2013, the inter-annual mean temperature remained relatively stable; however, the inter-annual precipitation varied greatly (Fig 1). According to the Naiman Meteorological Station, the mean annual temperatures were 5.9°C, 6.5°C, 5.6°C and 6.3°C in 2010, 2011, 2012, and 2013, respectively (Fig 1), whereas the corresponding annual precipitation was 347 mm, 200 mm, 491 mm, and 271 mm.

In this area, the geomorphologic landscape is characterized by sand dunes alternating with gently undulating inter-dune lowlands. The soils are light yellow in color, very infertile and sandy with a coarse texture and loose structure. In general, the maximum sand content (1–0.05 mm) in the soils of the study site is 90%, with organic matter and total N content of less than 1.0 g kg^-1^ and 0.13 g kg^-1^, respectively [22]. The vegetation is characterized by shrubland of *Artemisia halondendron* with scattered trees and windbreak tree belts of *Populus* spp. The dominant plant species include *A*. *halodendron*, *Caragana microphylla*, *Artemisia scoparia*, *Setaria viridis*, and *Phragmites australis* [23].

### Experiment design and treatments

The incubation experiment was performed in a relatively flat inter-dune lowland(the slope gradient is less than 2%) using the litterbag method from April 2010 to October 2013. In the selected grassland, 20 plots of 3×3 m^2^ were set up with a 1-m wide buffer zone around each plot. We used a randomized block design with each of five blocks including N addition (10 g·m^-2^·yr^-1^), water addition (100 mm·yr^-1^), coupling of N and water (NW treatment), and an untreated control (CK). Nitrogen was added as granular urea in April and July each year, whereas water addition was performed from mid-May to mid-September each year. We added 10 mm, 20 mm, 30 mm, 30 mm, and 10 mm of water to the designated plots each month from May to September to simulate an average increase in rainfall of approximately 30% in the corresponding period (Fig 1). Usually, 10 mm of water was added if an effective precipitation event (> 5 mm) did not occur within 7 days. Soil moisture (0–15 cm) was monitored twice each month using Time-Domain Reflectometry (TDR) from April to October each year.

In September 2009, plants of *A*. *halondendron*, *S*. *viridis*, and *P*. *australis* (represented by *Artemisia*, *Setaria* and *Phragmites*, respectively, in the following) were collected from the study area and taken to the laboratory. To avoid the heterogeneity in litter chemistry, all plants of the three species were sampled in a small area of dune grassland. We first collected the leaf material that had recently senesced but was still attached to the plant. Fine roots (<2.0 mm in diameter) of each plant were then cut from the crown after removing the soil by gentle washing with tap water. The root samples likely contained both live and recently dead roots. The leaves and fine roots were air-dried to a constant weight at room temperature, and then 5.0 g of each substrate was enclosed in 10×15 cm nylon net (1-mm mesh for leaves and 0.1-mm mesh for roots) bags. In April 2010, we separately placed leaf and root litterbags of each species on the surface and in the soil of the 20 plots. The leaf litterbags were fixed to the ground by metal pins to prevent movement, and root litterbags were vertically inserted into the soils at a depth of 10 cm. We sampled litterbags in April, July and October from 2010 to 2013 (except for April 2010 and July 2013 where litter bags were not sampled). A total of 400 litterbags (four treatments×five replicates×two litter types×ten sampling times) were prepared for each plant species. At each sampling time, 120 bags were collected from the 20 plots for three species, placed in polyethylene bags, and transported to the laboratory. In the laboratory, the leaf and root litter were removed from the bags, cleaned to remove any extraneous organic material, and weighed after drying at 60°C for 48 h. After the dry weight was measured, the samples were finely ground in a laboratory mill, and a portion of the litter was ashed to determine the ash-free dry weight of each sample. The ash-free dry mass was used directly or transformed for statistical analyses.

### Chemical analysis

The initial tissues and samples of roots and leaves obtained at each time point were dried at 70°C and finely ground in a laboratory mill before chemical analysis. The initial leaf and root samples were analyzed for C, N and lignin, whereas the subsequent samples were analyzed for N. Carbon was determined by the standard method of wet combustion [24]. Nitrogen was determined by the semi-micro Kjeldahl method [24], and lignin was determined via the detergent method [25].

### Data analysis

The decomposition constant, *k*, was determined using a single exponential decay model [26]:

ln(MtM0)=−kt,

where M_0_ is the initial mass, M_t_ is the mass remaining at time *t*, and *k* is the slope of this relationship. The *k* constant represents an integrated measure of decomposition over a given period of time. The remaining litter nitrogen was calculated by multiplying the sample mass by the respective nitrogen concentration. One-way ANOVA was performed to test the statistical significance of differences in initial litter chemistry, the decomposition constant (k) of each substrate in the four treatments. Four-way ANCOVA using a factorial design was performed to detect interactions of N addition, water addition, species and sampling time in mass remaining and nitrogen remaining for root and leaf litter, with block number as covariate. In the same way, the effects of N addition and water addition treatments on dry mass loss and nitrogen remaining were analyzed using two-way ANOVA models at each sampling time for each substrate. Prior to analysis, data were tested for normality using Shapiro-Wilk’s test. Response variables that did not show a normal distribution were log transformed. These analyses were performed using SPSS 16.0 for Windows (SPSS Inc., Chicago, Illinois).

## Results

### Soil moisture and initial litter chemistry

**Fig 2:**
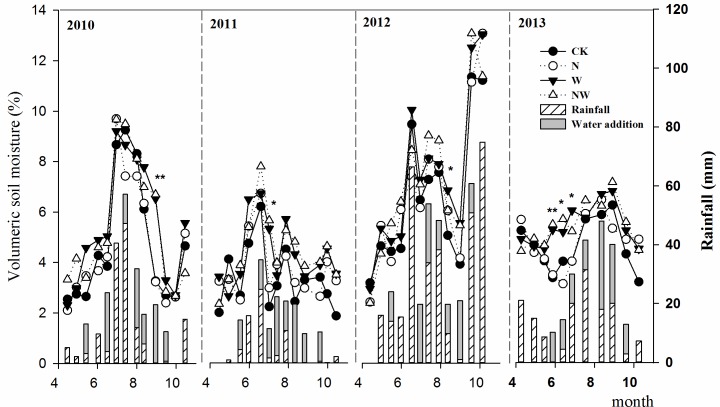
Soil moisture during the growth period from 2010 to 2013 in the four treatments: N addition, water addition, combined addition of N and water, and control. Key to abbreviations: N, nitrogen addition; W, water addition; NW, combined addition of nitrogen and water; CK, control. Significant differences in soil moisture among the treatments on each sampling date are indicated by *(*p*<0.05) and **(*p*<0.01).

Soil moisture (0–15 cm) in the selected dune grassland varied greatly but was closely correlated with the change in rainfall from 2010 to 2013 (Fig 2). One-way ANOVA indicated that water addition had significant effects on soil moisture only in the period of less natural rainfall and not in the rainy period. Over the entire experiment, significant differences in soil moisture among the four treatments were observed on only 6 of the 49 sampling dates.

The initial chemistry of the roots and shoots differed significantly among the three species (Table 1). Among the leaf litter, *Artemisia* had the highest C and N but lowest lignin concentrations, resulting in the lowest C:N and lignin:N values among the three species. Litter C was similar between *Setaria* and *Phragmites*, but litter N was higher in *Setaria* than in *Phragmites*, resulting in a lower C:N value for *Setaria*. Furthermore, the lignin concentration was significantly higher in *Phragmites* than in *Setaria*. Consequently, lignin:N was highest in *Phragmites* among the three species. In fine roots, *Setaria* roots exhibited the highest N concentration but lowest lignin content and C:N and lignin:N ratios, suggesting that *Setaria* root is a high-quality substrate, whereas *Artemisia* root was considered a low-quality substrate because it exhibited the highest lignin concentration but lowest N concentration among the three species. Overall, chemical analyses suggested a ranking in leaf litter quality for decomposition from *Artmisia* > *Setaria* > *Phragmites*, while for root litter the ranking would be *Setaria* > *Phragmites* > *Artemisia*.

**Table 1:** Initial leaf and root chemistry of the three dominant plant species in a desertified dune grassland in Northern China.

	C%	N%	Lignin%	P%	C:N	Lignin:N
Leaf						
*Artemisia*	44.6(0.8)a	1.83(0.04)a	6.3(0.3)a	0.31(0.01)a	25(0.9)a	3 (0.1)a
*Setaria*	42.3(0.7)ac	1.24(0.05)bh	10.5(0.3)bh	0.16(0.01)bg	34(1.4)afg	8 (0.3)bh
*Phragmites*	39.6(0.9)bc	0.96(0.04)c	15.7(0.7)ci	0.17(0.01)cg	42(2.3)bg	17(1.2)cg
Root						
*Artemisia*	49.2(0.8)d	0.6(0.0)di	17.3(0.3)di	0.19(0.01)dgh	89(6.1)c	31(2.0)d
*Setaria*	47.3(0.4)ae	1.15(0.03)eh	11.4(0.3)ehj	0.09(0.01)ei	39(2.0)dfg	10(0.7)eh
*Phragmites*	41.4(0.4)bce	0.72(0.02)fi	12.6(0.2)fj	0.12(0.00)fhi	58(1.3)e	18(0.4)fg

Note: Different lowercase letters in columns indicate significant differences at the 0.05 level in substrate of the three species. The values in parentheses are SE (*n* = 6).

### Dry mass loss

**Fig 3:**
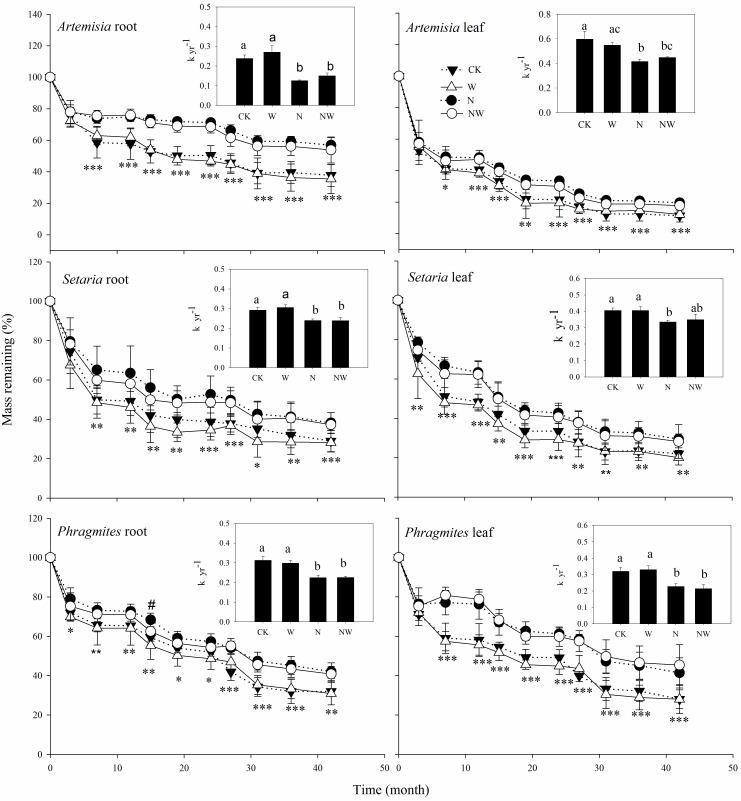
Dry mass remaining and decomposition rates in leaves and roots of the three species in the four treatments: N addition, water addition, combined addition of N and water, and control. The values are means±SE (*n* = 5). The results of two-way ANOVA (N addition and water addition as main effects) are shown on each sampling date for each litter type of the three species. Significant effects of nitrogen addition and water addition on dry mass loss is indicated by * and #, respectively, at each sampling time. * or # (*p*<0.05); **(*p*<0.01); ***(*p*<0.001). The abbreviations are as in Fig 2.

Fine roots decomposed more slowly than the leaf litter in the dune grasslands. After the 3.5-year incubation, on average, 89%, 78% and 72% of the initial leaf mass and 62%, 71% and 68% of the initial root mass (Fig 3) of *Artemisia*, *Setaria* and *Phragmites*, respectively, were lost in the control plots. Furthermore, ANOVA revealed that the decomposition rates of leaf litter and fine roots differed among the three species (*p*<0.001) (Table 2). For the leaf litter, the decomposition constant, *k*, of the three species in the control plots was approximately 0.6 (*Artemisia*), 0.41 (*Setaria*) and 0.32 (*Phragmites*), indicating that *Artemisia* leaves decomposed relatively quickly, whereas *Phragmites* leaves decomposed slowly. For the fine roots, *Artemisia* decomposed more slowly, whereas *Setaria* and *Phragmites* decomposed relatively quickly. The *k* values of *Setaria* and *Phragmites* (0.29 and 0.31) were similar but higher than that of *Artemisia* (0.24) in the control plots. These trends were totally consistent with the ranking in litter quality of leaves and roots, suggesting the decomposition rate in both leaves and roots was related to the initial litter N and lignin concentrations of the three species.

**Table 2:** *F*-values of four-way ANCOVA with block as covariate for the effects of N, water, species and sampling time on mass and nitrogen remaining (% of initial) in leaves and roots.

Source of Variation	Fine root		Leaf litter	
	Dry mass	N	Dry mass	N
Block	3.72	1.93	1.36	0.73
Nitrogen (N)	72.11***	10.49**	62.28***	74.53***
Water (W)	0.01	2.83	0.09	0.38
Species (S)	204.67***	189.53***	947.35***	1070.73***
Time (T)	213.75***	17.32***	395.25***	130.90***
N×W	0.26	0.87	0.43	0.09
N×S	25.46***	11.91***	22.66***	118.05***
N×T	2.13*	1.13	2.99**	4.77***
W×S	1.78	0.83	1.76	1.59
W×T	0.31	0.39	0.32	0.47
S×T	5.69***	1.76*	4.23***	12.65***
N×W×S	0.57	1.45	1.96	1.19
N×W×T	0.29	0.39	0.21	0.76
N×S×T	1.32	0.71	1.28	0.84
W×S×T	0.52	0.93	0.33	0.65
N×W×S×T	0.14	0.44	0.29	1.78*

Notes

*** *p*<0.001

** *p*<0.01

* *p*<0.05

Four-way ANOVA with a factorial design determined that dry mass loss in leaves and roots of the three species differed statistically significantly between sampling time points. The litter decomposition rates of leaves and roots were significantly negatively affected (*p*<0.001) by the addition of nitrogen but not water (Fig 3 and Table 2). Nitrogen addition reduced dry mass loss in leaves and roots by an average of 9% and 18% in *Artemisia*, 10% and 11% in *Setaria*, and 14% and 9% in *Phragmites* compared to the corresponding control treatments over the entire incubation period. The differences in the magnitude of the decline between leaves and roots among the three species indicate that the responses of mass loss to N addition varied within litter types and among species. Accordingly, the *k* values of leaves and roots significantly decreased (*p*<0.05) after N addition in *Artemisia*, *Setaria* and *Phragmites* compared with the control treatments (Fig 3), suggesting that N addition strongly inhibited litter decomposition in the dune grassland.

By contrast, we observed that water addition had little effect on litter decomposition in the dune grassland. Over the entire incubation period, significant effect of water addition on dry mass loss (p<0.05) was detected only at the fourth sampling time for *Phragmites* roots (Fig 3). The dry mass remaining in leaves between the water addition and control treatments was 12% and 11% in *Artemisia*, 20% and 22% in *Setaria*, and 28% and 28% in *Phragmites*, respectively, after the 3.5-year incubation but 35% and 38%, 28% and 29%, 31% and 32% in roots for the corresponding treatments and species. Furthermore, no significant differences were observed in *k* values of leaves and roots in the three species between the water addition and control treatments (Fig 3). In addition, the remaining masses of leaves and roots of the three species in the combined N and water treatment were consistently higher than those in the water addition treatment but similar to those in the N addition treatment during the entire incubation (Fig 3), suggesting that adding water did not influence the inhibitory effect of N addition on litter decomposition in the dune grassland.

### Nitrogen dynamics

**Fig 4:**
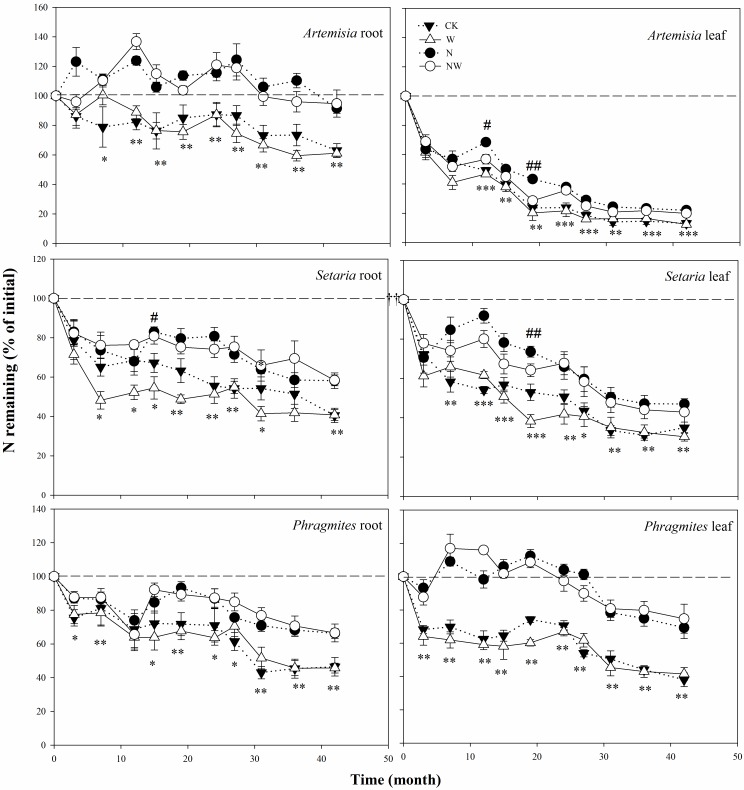
Nitrogen remaining (% of initial) in leaves and roots of the three studied species in the four treatments: N addition, water addition, combined addition of N and water, and control. The values are means±SE (*n* = 5). The results of two-way ANOVAs (N addition and water addition as main effects) are shown for each litter type of the three species on each sampling date. Significant effects of nitrogen addition and water addition on nitrogen remaining is indicated by * and #, respectively, at each sampling time. * or # (p<0.05); ** or ## (p<0.01); *** (p<0.001). A dashed line indicates the initial N value of 100%. The abbreviations are as in Fig 2.

**Fig 5:**
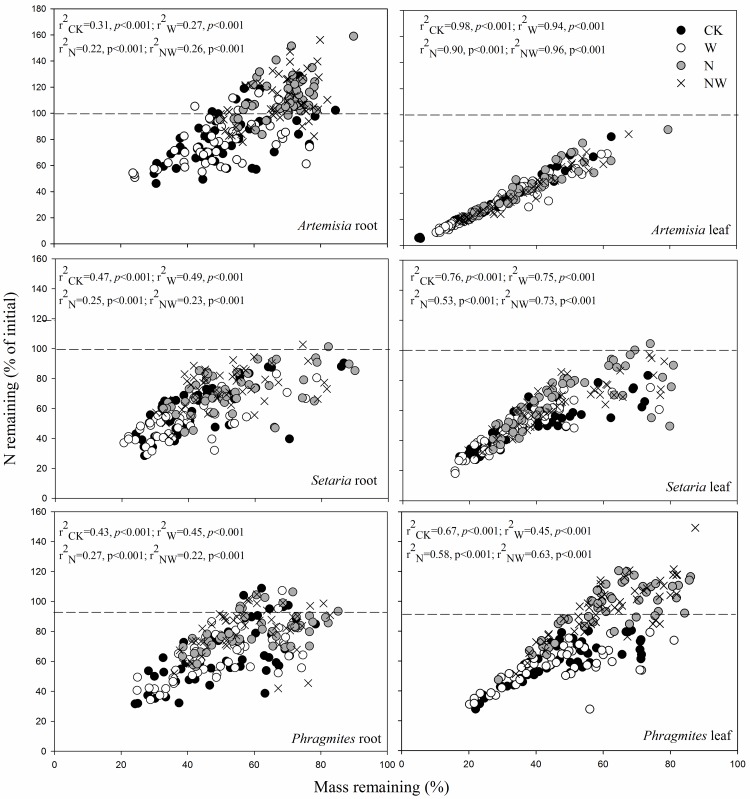
Nitrogen remaining (% of initial) vs. dry mass remaining for all replicates, harvests, and treatments in leaves and roots of the three studied species. The dashed line indicates the initial N value of 100%. The abbreviations are as in Fig 2.

Substantial N release was observed in the leaf and root litter of the three species during decomposition. Similar to the dry mass loss, N release from leaves and roots differed greatly among the three species (Table 2). For leaf litter, N release in *Artemisia* was relatively fast in the control plots, whereas N was released more slowly in *Phragmites* and *Setaria* (Figs 4 and 5). By contrast, in fine roots, more initial N was released in *Phragmites* and *Setaria* than in *Artemisia* in the control plots. After the 3.5-year incubation, the average N remaining among the three species was nearly 29% of initial N in the leaf litter but 50% of initial N in the fine roots, suggesting that nitrogen release was fast in leaf litter compared to fine roots.

Four-way ANOVA suggested that litter N remaining of the three species differed significantly between sampling time points. In addition, the N remaining in the leaf litter and fine roots was also strongly affected (*p*<0.001) by N addition but not by water addition (Table 2). Similar to the dry mass loss, N release in leaves and roots was significantly inhibited by N addition alone or in combination with water. However, the inhibitory effect of supplemental N on litter N release was not straightforward; the species and litter types responded differently, and the response also changed with time (Fig 4). On average, supplemental N inhibited litter N release in leaves and roots by 10% and 33% in *Artemisia*, 12% and 18% in *Setaria*, and 35% and 15% in *Phragmites*, respectively, relative to the control treatments over the entire incubation period. Furthermore, we observed net N immobilization in *Artemisia* roots and *Phragmites* leaves on some sampling dates (Fig 4) after adding N. Over the entire experiment, neutral effects of water addition on N release were observed for both leaves and roots of the three species despite several exceptions in *Artemisia* and *Setaria* (Fig 4).

## Discussion

### Litter chemistry and litter types

As expected, we observed significant differences in dry mass loss and nitrogen release in leaf litter and fine roots among the three species in the present study. With higher N content and lower C/N and lignin content in leaf litter but lower N content and higher C/N and lignin content in fine roots (Table 1), *Artemisia* had the highest leaf decomposition rate and the lowest root decomposition rate among the three species over the entire incubation period. Additionally, nitrogen release from *Artemisia* was relatively fast in leaf litter and slow in fine roots among the three species. These results suggest that the decomposition rate in both leaves and roots was related to the initial litter N and lignin concentrations of the three species. This conclusion is in agreement with previous observations that high-quality litter (characterized by higher N concentrations, lower C/N ratios and lignin content) decomposes faster than low-quality litter [27, 28].

Contrary to our hypothesis, dry mass loss and N release were more rapid in leaves than in roots for the three species studied here, suggesting that the lower frequency of moisture oscillations in the soil relative to on the ground hardly facilitate root decomposition in this area. A slow decomposition rate and nutrient release in roots compared to leaves are often ascribed to the higher lignin and C/N of roots [29, 30]. In the present study, this relationship can explain the more rapid loss of dry mass in leaf litter than in roots of *Artemisia* but may not hold for *Setaria* and *Phragmites*. *Setaria* leaves decomposed more quickly than roots (decomposition constant *k* in control plot were 0.41 for leaves and 0.29 for roots) while no significant differences (p<0.05) were detected in N and lignin contents between roots and leaves. Further, decomposition rates in leaves and roots of *Phragmites* were very close (decomposition constant *k* in control plot were 0.32 for leaves and 0.31 for roots) but *Phragmites* had significantly (*p*<0.05) higher lignin and N contents in leaves than in roots (Table 1). The loss of dry mass and nutrients may also have been faster in leaves than in roots due to leaf photodegradation on the soil surface [31, 32], differences in microbial decomposer communities on the surface and in the soil [33], and differences in the concentrations of other initial organic components, such as soluble carbohydrates and holocellulose, between leaf litter and fine roots [34]. Further studies are needed to determine which factors mainly contribute to the more rapid loss of dry mass in leaves than in roots in this system.

### Effects of supplementary nitrogen and water on mass loss

The decomposition rates in the combined N and water treatment were consistently higher than those in the water addition and control treatments but similar to those in the N addition treatment for both leaves and roots of the three species during the entire incubation (Fig 1), suggesting that there was no interactive effect of combining water and nitrogen on litter decomposition.

Contrary to our hypothesis, litter decomposition was not stimulated but was apparently inhibited after adding N to the soils of the dune grassland. This result is in contrast to previous findings that nitrogen addition stimulates litter decomposition [30, 35] but supports reports in which negative effects of the addition of N to soils on litter decay were observed [36]. Knorr et al. [16] suggested that N addition generally stimulated the decomposition of high-quality litter while suppressing the decomposition of low-quality litter. In the present study, we observed significant inhibitory effects of added N on decomposition rates of either low-quality litter (e.g., *Phragmites* leaf and *Artemisia* root) or higher-quality litter (e.g., *Artemisia* leaf and *Setaria* root), but the magnitude of the mass loss of leaves and roots with added N varied between the low-quality litter and high-quality litter. Added N suppressed litter decomposition rates by 9% in *Artemisia* leaves (high-N and low-lignin litter) but 14% in *Phragmites* leaves (low-N and high-lignin litter). Accordingly, the mass losses of *Setaria* roots (high quality) and *Artemisia* roots (low quality) were reduced by 11% and 18% after adding N. This pattern implies that the effects of N inhibition on litter decomposition may be related to litter quality.

Although some evidence suggests that nitrogen addition inhibits litter decomposition, the characteristic of inhibition by N enrichment remains unclear. Several explanations of the decrease in the decomposition rate after adding N to soils have been proposed, and a decrease in microbial activity upon the addition of N to soil is widely accepted as the primary factor inhibiting litter decomposition. A study in a semiarid grassland [37] suggested that both microbial biomass and microbial respiration rates are reduced by N fertilization. A reduction of microbial activity by N addition was also observed in the Harvard forest [38] and in a temperate hardwood and pine forest [39]. However, we observed that soil respiration rates were very slightly stimulated by N fertilization in previous studies at the same site [40], indicating that microbial activity may not have been suppressed in our study. Another prevailing explanation for the decrease in the decomposition rate upon N addition is the inhibition of lignin-degrading enzyme activity. Added N reduces the activity of microbial extracellular enzymes responsible for the breakdown of lignin, causing high-lignin litter types to respond more negatively to N additions than more labile litter types [36, 41, 42]. This phenomenon appeared to occur in the present study because the magnitudes of the decrease in mass loss in substrates with high lignin contents (e.g., *Phragmites* leaf and *Artemisia* root) were larger than those in substrates with low lignin contents (e.g., *Artemisia* leaf and *Setaria* root) after adding N.

We observed neutral effects of water input on mass loss in both leaves and roots in the present study, suggesting that the decomposition processes in this desertified dune grassland are not as closely regulated by water as anticipated. Our results are in agreement with evidence that the mass loss of litter is unrelated to precipitation or actual evapotranspiration in some arid ecosystems. Many previous studies have demonstrated that supplemental water has little effect on mass loss in desert ecosystems [13, 43]. However, some opposing evidence suggested that litter decomposition rates were positively correlated with incoming annual precipitation in semiarid ecosystems [2, 44]. The effect of precipitation on litter decomposition is primarily ascribed to decomposer activity controlled by water availability, particularly in water-limited systems. However, soil microorganisms have different thresholds of soil water availability, and some resistant groups may maintain vigorous activities even at low water availability [45, 46]. This activity may explain the neutral effects of water input on litter decomposition in the present study. In addition, some reports pointed out that the effect of water availability on decomposition differed between aboveground and belowground decomposition [8, 17]. In contrast to these findings, we observed similar responses of mass loss to water input between aboveground leaves and belowground roots. This discrepancy may reflect the low water-holding capacity of the soils in the dune grassland at our study site. Water addition temporarily improved the soil water status only in the period of natural drought, and no significant differences were observed between water addition treatment and control on most of the sampling dates (Fig 2). Accordingly, significant effects of water addition on mass loss and N release were detected at several sampling times where natural drought occurred (Figs 3 and 4). This implies that litter decomposition in semi-arid dune grassland may be restricted only during drier period, thus water addition during the period of normal rainfall has little effect on decomposition.

### Nitrogen dynamics during decomposition

Nitrogen is considered a key limiting nutrient for the growth of decomposer populations in plant litter and is usually immobilized or mineralized by microorganisms in terrestrial ecosystems during decomposition. Previous studies have suggested that N immobilization or mineralization in decomposing litter is predominantly controlled by the C:N ratio of the substrate, and the net N loss usually occurs in decomposing litter when the C:N ratio is less than the critical threshold of 5 to 15 [47, 48]. In the present study, however, net N immobilization did not occur during the decomposition of leaf litter and fine roots (in the control plots), although our results demonstrated that the C:N ratios of the leaf litter and fine roots of the three species were all higher than 15 (Table 1), suggesting that using the initial litter C:N ratio hardly estimates the occurrence of net N immobilization or mineralization during litter decomposition in this system. Other factors, such as differences in physicochemical environments or decomposer composition, may control N immobilization or mineralization of decomposing litter in dune grassland. Moreover, the net release of N from leaves and roots of plant species may have a significant effect on N cycling and thus rapidly improve the soil N availability in the desertified dune grassland.

N addition, both alone and in combination with water, appeared to inhibit N release in our study, whereas water input had no effect on N loss. This result implies that environmental changes in enhanced N deposition could potentially improve nitrogen retention in desertified dune grasslands in northern China. Furthermore, we observed shifts from net N release to net immobilization in substrates of low quality (low N and high lignin) (e.g., *Phragmites* leaf and *Artemisia* root) on some sampling dates (Fig 4). Hobbie [49] also reported that litter with the lowest concentrations of N immobilized the most N, which was mainly ascribed to the slow mass losses of these substrates after N addition. In the present study, dry mass and N were lost in a proportional manner (Fig 5). The remaining dry mass can explain approximately 50–95% of the total variance of N remaining in leaf litter for all treatments but approximately 20–50% of the total variance of N remaining in fine roots, suggesting that other factors may contribute to N loss in belowground roots.

## Conclusions

The decomposition rate in both leaves and roots was related to the initial litter N and lignin concentrations of the three species. However, litter quality did not explain the slower mass loss in roots than in leaves in the present study, suggesting that further research is necessary to elucidate the factors that control decomposition in roots and leaves in desertified dune grassland. Nitrogen addition, either alone or in combination with water, inhibited dry mass loss in the leaves and roots of the three species, whereas water input had little effect on the decomposition of leaf litter and fine roots. These results indicate that a negative effect of atmospheric N deposition on decomposition will stimulate ecosystem C sequestration in dune grasslands, unless rapid decomposition of litter with higher N concentrations subject to relatively high N deposition offsets this inhibitory effect. In particular, the magnitude of N inhibition was lower for high-lignin litter than for low-lignin litter in this study. Net N release was observed in the leaves and roots of the three species, but N addition suppressed N release and even shifted net N release to net immobilization in substrates with low quality. This implies that atmospheric N deposition may improve nitrogen retention in desertified dune grassland in northern China.
